# Exercise-Induced Atrial Remodeling in Female Amateur Marathon Runners Assessed by Three-Dimensional and Speckle Tracking Echocardiography

**DOI:** 10.3389/fphys.2022.863217

**Published:** 2022-07-04

**Authors:** Zofia Lasocka, Zuzanna Lewicka-Potocka, Anna Faran, Ludmiła Daniłowicz-Szymanowicz, Radosław Nowak, Damian Kaufmann, Anna Kaleta-Duss, Leszek Kalinowski, Grzegorz Raczak, Ewa Lewicka, Alicja Dąbrowska-Kugacka

**Affiliations:** ^1^ Department of Cardiology and Electrotherapy, Medical University of Gdańsk, Gdańsk, Poland; ^2^ First Department of Cardiology, Medical University of Gdańsk, Gdańsk, Poland; ^3^ Institute for Radiology, Cantonal Hospital Aarau, Aarau, Switzerland; ^4^ Department of Medical Laboratory Diagnostics—Fahrenheit Biobank BBMRI.pl, Medical University of Gdańsk, Gdańsk, Poland; ^5^ BioTechMed Centre/Department of Mechanics of Materials and Structures, Gdańsk University of Technology, Gdańsk, Poland

**Keywords:** marathon running, atrial remodeling, 3D echocardiography, 2D speckle-tracking echocardiography, female amateur athletes, endurance training

## Abstract

Endurance athletes have an increased risk of atrial remodeling and atrial arrhythmias. However, data regarding atrial adaptation to physical exercise in non-elite athletes are limited. Even less is known about atrial performance in women. We aimed to elucidate exercise-induced changes in atrial morphology and function in female amateur marathon runners using three-dimensional (3D) echocardiography and two-dimensional (2D) speckle tracking echocardiography (STE). The study group consisted of 27 female (40 ± 7 years) amateur athletes. Right (RA) and left atrial (LA) measures were assessed three times: 2–3 weeks before the marathon (stage 1), immediately after the run (stage 2), and 2 weeks after the competition (stage 3). Directly after the marathon, a remarkable RA dilatation, as assessed by RA maximal volume (RAVmax, 31.3 ± 6.8 vs. 35.0 ± 7.0 ml/m^2^; *p* = 0.008), with concomitant increase in RA contractile function [RA active emptying fraction (RA active EF), 27.7 ± 8.6 vs. 35.0 ± 12.1%; *p* = 0.014; RA peak atrial contraction strain (RA PACS) 13.8 ± 1.8 vs. 15.6 ± 2.5%; *p* = 0.016] was noticed. There were no significant changes in LA volumes between stages, while LA active EF (34.3 ± 6.4 vs. 39.4 ± 8.6%; *p* = 0.020), along with LA PACS (12.8 ± 2.1 vs. 14.9 ± 2.7%; *p* = 0.002), increased post race. After the race, an increase in right ventricular (RV) dimensions (RV end-diastolic volume index, 48.8 ± 11.0 vs. 60.0 ± 11.1 ml/m^2^; *p* = 0.001) and a decrease in RV function (RV ejection fraction, 54.9 ± 6.3 vs. 49.1 ± 6.3%; *p* = 0.006) were observed. The magnitude of post-race RV dilatation was correlated with peak RA longitudinal strain deterioration (r = −0.56, *p* = 0.032). The measured parameters did not differ between stages 1 and 3. In female amateur athletes, apart from RV enlargement and dysfunction, marathon running promotes transient biatrial remodeling, with more pronounced changes in the RA. Post-race RA dilatation and increment of the active contraction force of both atria are observed. However, RA reservoir function diminishes in those with post-race RV dilation.

## Introduction

Physical activity is gaining popularity in the general population, with a growing number of both male and female amateur athletes. Endurance training promotes structural, electrical, and functional changes in the heart, with significant gender discrepancies (Di Paolo and Pelliccia, 2007; Pelliccia and Adami, 2017). Until the first half of the 20th century, women were considered physiologically unable to perform sports at a competitive level, and most publications on exercise-induced cardiac remodeling pertain to male athletes. Recently, however, the participation of women in marathons increased from 10% in 1980 to 43% in 2013 (Roberts, et al., 2013). Despite the growing number of female athletes, little is known about the long-term cardiac effects of prolonged exercise in this population.

Traditionally, the ventricles are the main focus of clinical research, but the importance of atrial morphology and function should not be underestimated. Exercise-induced atrial dilatation, as a physiologic adaptation to exercise conditioning, has been previously described (Pelliccia et al., 2005; Brugger et al., 2014). According to Gabrielli et al. (2014), the atria of athletes function at lower strain, larger volumes, and higher atrial wall stress. Indeed, the evaluation of atrial performance plays a fundamental role in the assessment of an athlete’s heart, differentiating physiologic adaptation to exercise from pathologic changes (D'Ascenzi et al., 2018). Recent reports have shown that exercise-induced atrial remodeling may contribute to development and perpetuation of atrial fibrillation (AF) (Abdulla and Nielsen, 2009; Aizer et al., 2009; Mont et al., 2009). However, the aforementioned outcomes regard mainly male athletes, with scarce data on atrial adaptation to endurance training in women (Pelliccia et al., 2005; D'Ascenzi et al., 2014).

The majority of studies describing atrial volumes and function in athletes are based on two-dimensional (2D) echocardiographic assessment, but 2D methods rely strongly on correct positioning and angulation of imaging planes, complicated in the case of atria. Real-time, three-dimensional (3D) echocardiography may overcome all limitations of conventional echocardiography, without any angulation issues or geometric assumptions about atrial shape (Artang et al., 2009; Caselli et al., 2010).

In addition, speckle tracking echocardiography (STE) allows objective and quantitative evaluation of global and regional atrial deformation. The atrial longitudinal 2D strain is considered the most useful parameter for functional analysis of both atria due to its significant feasibility and reproducibility (Padeletti et al., 2012). An accurate assessment of atria in endurance athletes is crucial to provide a comprehensive evaluation of biatrial remodeling and its relation to exercise capacity (Nielsen et al., 2021).

Therefore, the aim of our study was to assess echocardiographic parameters of biatrial performance at rest and directly after the marathon run in female amateur athletes and investigate whether this prolonged training correlates with improvement or worsening of atrial function. We hypothesized that endurance of physical activities, such as marathon run, may lead to corresponding cardiovascular changes in amateurs, as in elite athletes.

## Materials and Methods

### Study Design and Participants

We enrolled 27 female Caucasian marathon runners aged between 28 and 57 years, who participated in the XXIV Orlen Solidarity and 5th Gdansk Marathon. The participants were healthy, in sinus rhythm, without cardiovascular comorbidities or other chronic diseases, and had a negative family history of cardiac disease or sudden cardiac death.

The study protocol consisted of three stages: 2–3 weeks before the start of the race (stage 1), immediately after the marathon run, on the finish line (stage 2), and 2 weeks after the competition (stage 3). At each stage, physical examination with anthropometric data (height, weight, body mass index, and blood pressure), electrocardiography (ECG), and transthoracic echocardiography was performed. Furthermore, baseline assessment included the intensity of training, defined in hours and distance per week; number of completed marathons; cardiopulmonary exercise test on a treadmill; and 24-h Holter ECG monitoring. The index race performance time was also noted. During the competition, participants were allowed to rehydrate *ad libitum*, and no food intake restrictions were advised. Detailed study information was provided to all volunteers, and written consent was obtained from all participants prior to the study. The study protocol was approved by the Bioethics Committee of the Medical University of Gdansk, Poland (No. NKBBN 104/2016).

### 3D Echocardiographic Assessment

**FIGURE 1:**
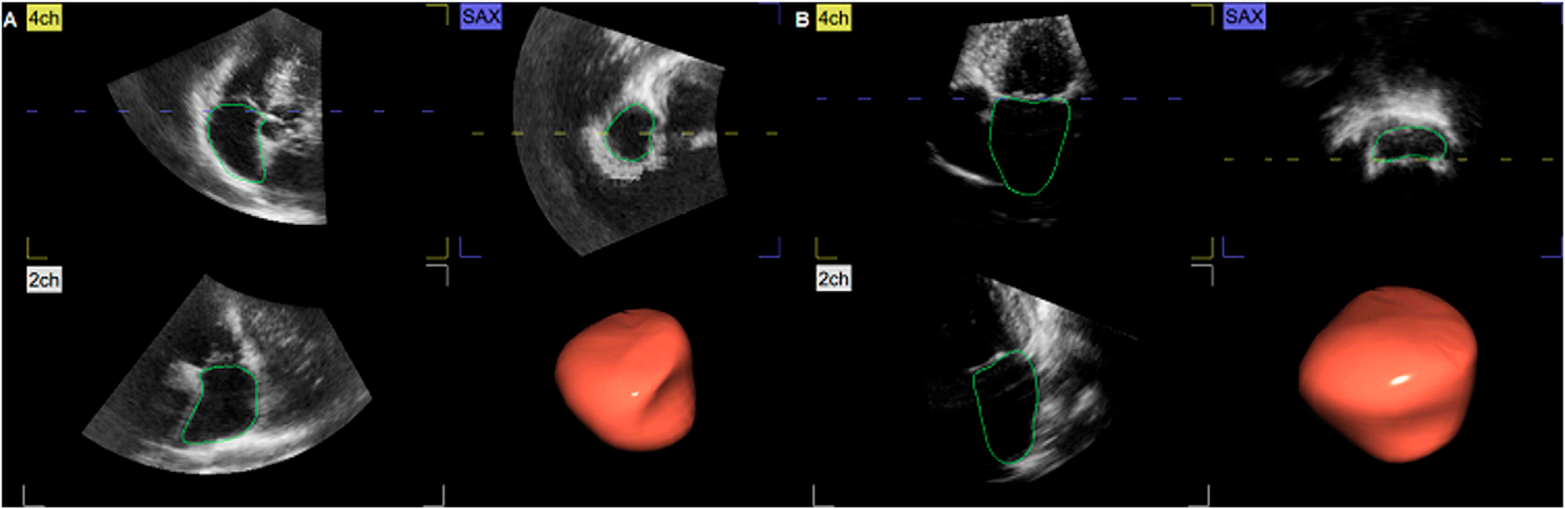
Three-dimensional (3D) echocardiographic assessment of the right atrium (RA) **(A)** and left atrium (LA) **(B)**. From the apical view, both transversal and longitudinal visualization of atrial volume changes during the cardiac cycle was obtained, with endocardial contouring and cast reconstruction. 2ch, two-chamber view; 4ch, four-chamber view; SAX, short-axis view.

Standard and 3D echocardiographic examination was performed using a commercially available system (Vivid E9 and E95, GE Healthcare, Horten, Norway), following the current recommendations of the European Association of Preventive Cardiology (EAPC) and European Association of Cardiovascular Imaging (EACVI) (Kou et al., 2014; Pelliccia et al., 2018). The subjects were studied in the steep left-lateral position. Data sets of both ventricles and atria were obtained from the apex from six cardiac cycles, by means of electrocardiographically gated full-volume 3D echocardiography. Off-line data analysis was performed by two researchers, using echocardiographic quantification software (EchoPac 201, GE Healthcare, Norway). The blood–tissue interface was automatically initialized by the software and afterward manually corrected frame-by-frame by tracing the endocardium from the 2-, 3-, and 4- chamber and short-axis view. The atrial data set alignment, identifying atrial volumes throughout the cardiac cycle, is demonstrated in Figure 1.

To quantify ventricular 3D morphology, we determined ventricular end-diastolic volume (EDV) and ventricular end-systolic volume (ESV), indexed to body surface area (BSA). In the case of left ventricular (LV) function, indexed LV stroke volume (LVSV) and LV ejection fraction (LVEF) were measured. While considering right ventricular (RV) contractility, RV ejection fraction (RVEF) and RV fractional area change (RVFAC) were calculated.

**FIGURE 2:**
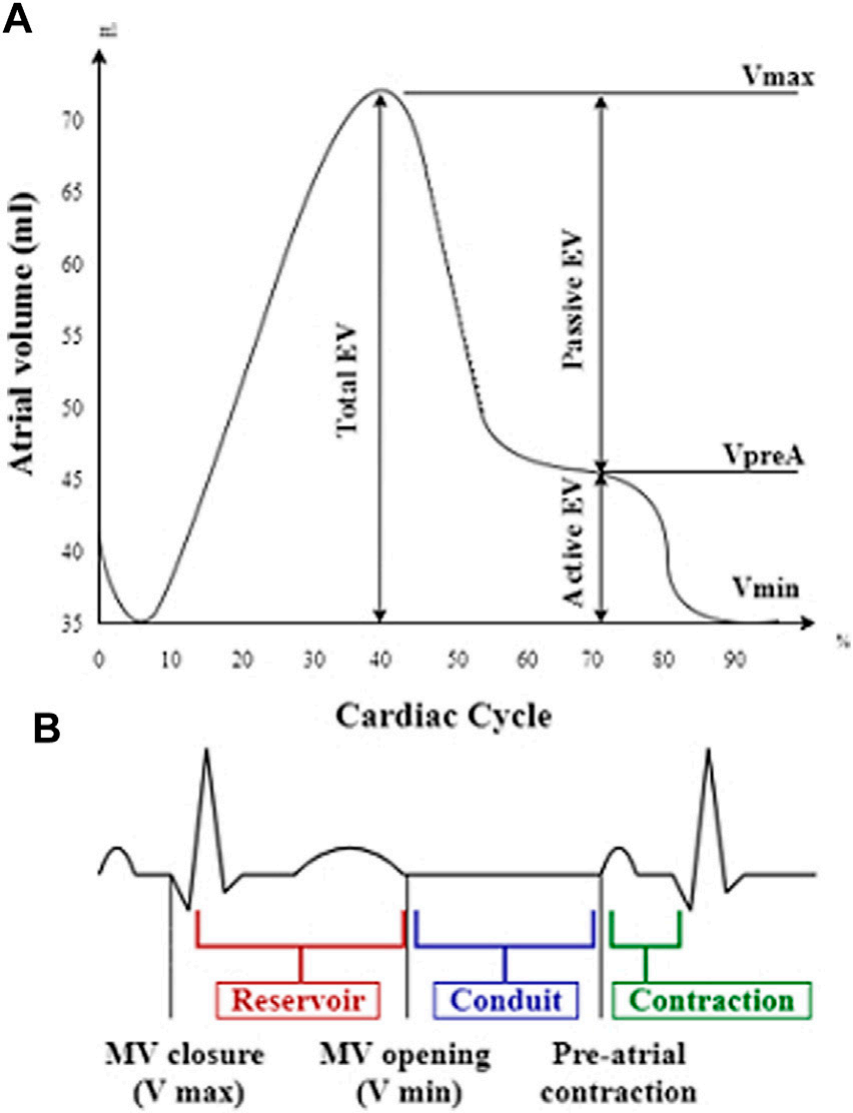
Atrial volume–time curve presenting atrial volume changes during the cardiac cycle **(A)** in relation to the reservoir, conduit, and contraction phases on electrocardiogram **(B)**. EV, emptying volume; Vmax, maximal volume; VpreA, pre-A-wave volume; Vmin, minimal volume; MV, mitral valve.

Volumetric changes of both atria were measured at three stages: maximal volume (Vmax, at the end of ventricular systole just before mitral valve opening), minimal volume (Vmin, at the end of ventricular diastole just before mitral valve closure), and atrial volume before contraction (VpreA). The atrial volumes were indexed to BSA. Total emptying volume (EV) was defined as Vmax-Vmin. Using these volumetric data, we computed the total emptying fraction (EF) as ([Vmax-Vmin]/Vmax) x 100, the passive EF as ([Vmax-VpreA]/Vmax) x 100, and the active EF as ([VpreA-Vmin]/VpreA) x 100, as parameters of the atrial reservoir, conduit, and contraction function, respectively (Figure 2). According to the current EAPC/EACVI guidelines (Pelliccia et al., 2018), the normal ranges for left atrial (LA) volume are 36 ml/m^2^ in men and 33 ml/m^2^ in women, while the upper limits for right atrial (RA) volume are 33.8 ml/m^2^ in men and 29.3 ml/m^2^ in women.

### 2D Speckle Tracking Echocardiography

Atrial myocardial deformation was measured with the use of 2D STE during breath-holding with a stable ECG tracing. Three consecutive heart cycles were recorded and averaged. The frame rate was set between 60 and 80 frames per second. LA strain parameters were obtained from the apical 4-chamber and 2-chamber views, whereas RA strain was calculated from the apical 4-chamber view. The endocardial surface of both atria was manually traced by a point-and-click approach. Then, the epicardial surface tracing was automatically generated by the system, creating a region of interest (ROI). After manual adjustment of ROI, the software automatically divided the atrial wall into six equidistant segments and analyzed it.

**FIGURE 3:**
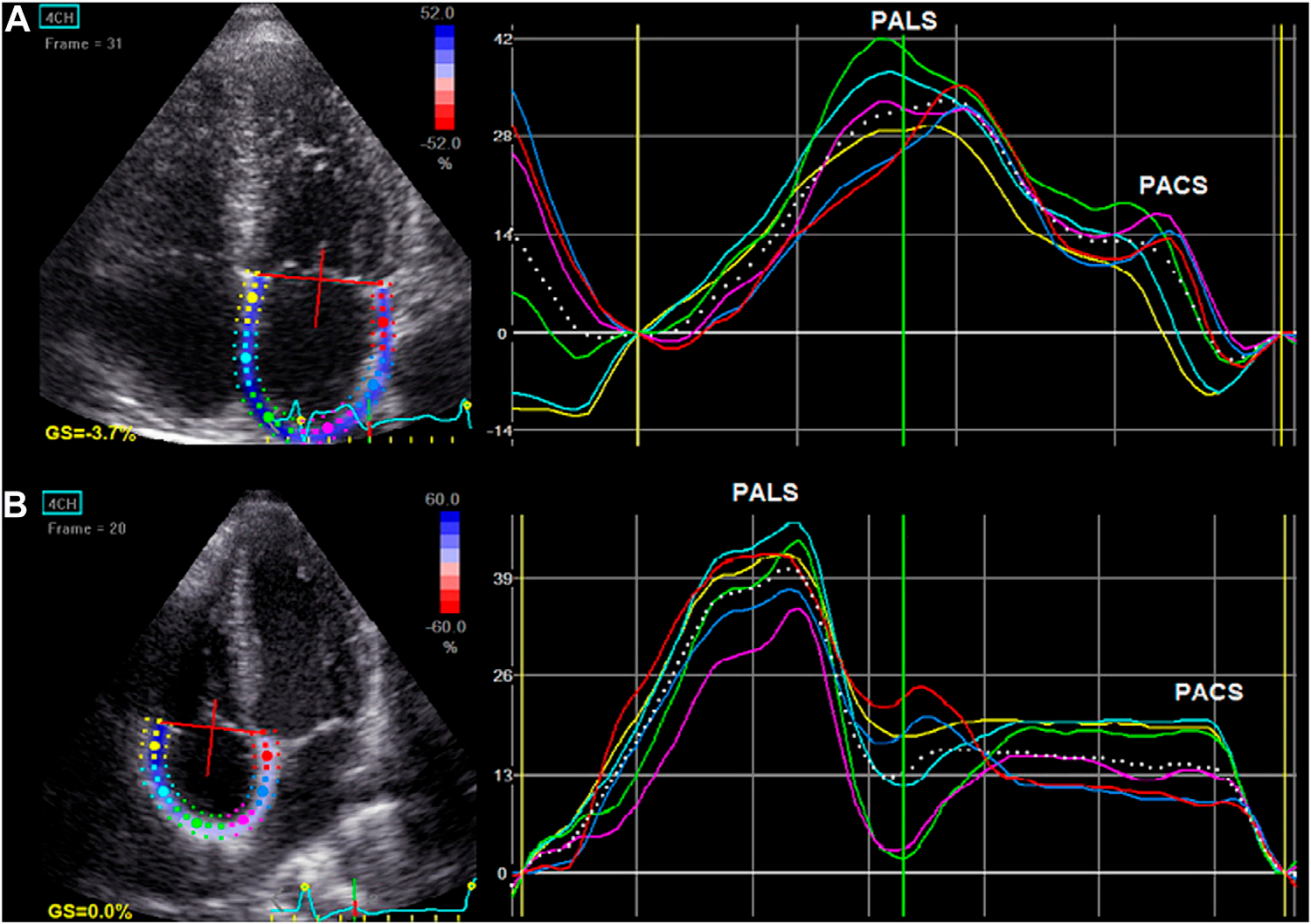
Left atrial **(A)** and right atrial **(B)** longitudinal myocardial deformation by two-dimensional speckle tracking echocardiography (2D STE). The dashed curve depicts the average strain. The first positive peak of the curve is the peak atrial longitudinal strain (PALS), measured at the end of the reservoir phase. The second peak, just before the active atrial contraction, represents peak atrial contraction strain (PACS).

Finally, the longitudinal strain curves for each segment and a mean curve of all segments were generated (Figure 3). Before the opening of the mitral valve, when the atrium fills and stretches until its peak in systole, positive atrial reservoir strain (peak atrial longitudinal strain, PALS) was measured. After the opening of the mitral valve, the atrium empties quickly and shortens, and the strain decreases up to a plateau, followed by a second positive peak, which corresponds to the atrial contractile phase (peak atrial contraction strain, PACS). Reference values for LA and RA strain parameters were used according to current recommendations (Padeletti et al., 2012; Nielsen et al., 2021).

### Statistical Analysis

Data analysis was performed using Statistica 13.3 software (Statsoft Inc., Tulsa, Oklahoma, United States). The normal distribution of all continuous variables was examined using the Shapiro–Wilk test, and data were presented as mean ± standard deviation (SD). The comparison between the three stages was performed with repeated measures ANOVA analysis and the post-hoc Tukey test for normally distributed data. Non-normally distributed measurements were compared with Friedman ANOVA and post-hoc for Friedman ANOVA. Post-hoc analysis was performed for stages 1 and 2 and 1 and 3. In addition, the generalized eta-squared was provided as the recommended parameter when reporting effect sizes for ANOVA and Kendall’s W for the parameters where Friedman’s ANOVA test was applied (Supplementary Material). Correlations were measured using the Spearman and Pearson method, as appropriate for data distribution. A *p*-value < 0.05 was considered statistically significant.

## Results

### Baseline Characteristics

All participants completed the marathon; however, due to poor imaging, both pre- and post-race RA measures were obtainable only in 16 runners. LA parameters were successfully assessed in the whole study group. Basic demographics and morphometric data of the marathoners are listed in Table 1. The mean age of 27 female amateur marathon runners was 40 ± 7 years. As a parameter of aerobic capacity, maximal oxygen uptake (VO_2_max) was measured at stage 1, and it reached 42.8 ± 5.1 ml/kg/min. Systolic and diastolic blood pressure values were within the normal ranges, both at rest and after the run. The female athletes completed 5.3 ± 4.0 marathons, with a current average training duration of 8.1 ± 3.5 h per week and mean running distance of 60.7 ± 27.8 km per week. The marathon performance time reached 253.0 ± 32.5 min.

**TABLE 1:** Physical characteristics and intensity of training of the studied group.

Variable	Women (N = 27)
Age, years	40 ± 7
Weight, kg	59 ± 8
Height, cm	166 ± 5
BMI, kg/m^2^	22 ± 3
BSA, m^2^	1.65 ± 0.11
VO_2_max, ml/kg/min	42.8 ± 5.1
Training intensity	
Hours of running/week	8.1 ± 3.5
Distance running/week, km	60.7 ± 27.8
Number of completed marathons	5.3 ± 4.0
Marathon performance time, min	253.0 ± 32.5

BMI, body mass index; BSA, body surface area; VO2max, maximal oxygen uptake.

In 24-h Holter monitoring, the underlying baseline rhythm was sinus with a minimal heart rate (HR) of 40.3 ± 4.4 bpm, maximal HR of 136.8 ± 18.6 bpm, and average HR of 64.0 ± 6.3 bpm. Both premature supraventricular complexes (SVPCs) and premature ventricular complexes (PVCs) were rarely observed within the recording time.

### Echocardiographic Measurements

Table 2 summarizes exercise-induced 3D echocardiographic changes of RV and LV. After the marathon, there was a significant increase in the RV, with a decrease in LV dimensions. RV systolic contractility, assessed by RVEF and RVFAC, significantly decreased at stage 2, while LVEF did not differ remarkably post race. We did not notice any remarkable differences in the RV or LV parameters between stage 1 and stage 3.

**TABLE 2:** Biventricular measures assessed by three-dimensional echocardiography in female amateur athletes.

Parameter	Female (N = 27)			ANOVA *p*-Value	Post-Hoc *p*-Value	
	Stage 1	Stage 2	Stage 3		S1 vs. S2	S1 vs. S3
LVEDV index, mL/m^2^	66.2 ± 7.9	60.1 ± 9.5	65.3 ± 7.5	<0.001	<0.001	0.278
LVESV index, mL/m^2^	25.4 ± 4.3	22.4 ± 4.2	24.8 ± 3.6	<0.001	<0.001	0.480
LVSV index, mL/m^2^	40.8 ± 5.8	36.9 ± 7.5	40.4 ± 5.7	<0.001	<0.001	0.396
LVEF, %	61.6 ± 4.5	62.8 ± 3.7	62.1 ± 3.7	0.059	-	-
RVEDV index, mL/m^2^	48.8 ± 11.0	60.0 ± 11.1	51.5 ± 8.3	0.002	0.001	0.402
RVESV index, mL/m^2^	22.2 ± 6.5	30.7 ± 7.0	23.8 ± 5.0	<0.001	<0.001	0.310
RVSV index, mL/m^2^	26.6 ± 6.4	29.3 ± 6.1	27.8 ± 6.0	0.468	-	-
RVEF, %	54.9 ± 6.3	49.1 ± 6.3	54.3 ± 5.9	0.004	0.006	0.869
RVFAC, %	47.2 ± 5.9	42.6 ± 5.3	48.2 ± 2.9	0.009	0.037	0.897

LVEDV, left ventricular end-diastolic volume; LVESV, left ventricular end-systolic volume; LVSV, left ventricular stroke volume; LVEF, left ventricular ejection fraction; RVEDV, right ventricular end-diastolic volume; RVESV, right ventricular end-systolic volume; RVSV, right ventricular stroke volume; RVEF, right ventricular ejection fraction; RVFAC, right ventricular fractional area change. Data are presented as mean ± standard deviation.

A comparison of echocardiographic parameters of LA between stages is shown in Table 3. When analyzing the atrial size, there were no significant changes in 3D LA volumes between the stages, which, however, have tendency to decrease after the marathon run. Taking present EAPC/EACVI recommendations (Pelliccia et al., 2018) into consideration, LA enlargement was observed at baseline in 48% of female athletes, in 41% after the training, and in 44% of study participants at stage 3 (no differences between the stages). Functional measures of LA, such as LA total EF and LA passive EF, remained within the same range, while LA active EF increased post race (34.3 ± 6.4 vs. 39.4 ± 8.6%; *p* = 0.020). The outcomes were consistent with 2D STE values. Regarding LA reservoir function, there were no significant differences in LA PALS between stages, while the active phase of atrial contraction increased immediately after the marathon (LA PACS, 12.8 ± 2.1 vs. 14.9 ± 2.7%; *p* = 0.002) and returned to baseline during the recovery period.

**TABLE 3:** Left atrial parameters assessed by three-dimensional and two-dimensional speckle tracking echocardiography in female amateur athletes.

	Female (N = 27)				Post-Hoc *p*-Value	
Parameter	Stage 1	Stage 2	Stage 3	ANOVA *p*-Value	S1 vs. S2	S1 vs. S3
LAVmax index, mL/m^2^	32.7 ± 6.8	31.2 ± 8.6	32.7 ± 7.0	0.314	-	-
LAVmin index, mL/m^2^	14.0 ± 3.8	12.7 ± 4.0	13.7 ± 3.9	0.056	-	-
LAVpreA index, mL/m^2^	21.4 ± 5.8	21.1 ± 6.5	20.9 ± 5.6	0.820	-	-
LA total EV index, mL/m^2^	18.9 ± 4.2	18.8 ± 5.6	19.1 ± 3.8	0.893	-	-
LA passive EV index, mL/m^2^	11.4 ± 3.0	10.4 ± 3.9	12.0 ± 3.0	0.086	-	-
LA active EV index, mL/m^2^	7.5 ± 2.5	8.4 ± 3.4	7.0 ± 2.5	0.087	-	-
LA total EF, %	57.8 ± 5.5	59.8 ± 5.7	58.4 ± 5.2	0.116	-	-
LA passive EF, %	35.4 ± 8.1	33.2 ± 8.2	36.0 ± 8.7	0.158	-	-
LA active EF, %	34.3 ± 6.4	39.4 ± 8.6	34.0 ± 7.5	0.010	0.020	0.995
LA PALS, %	37.4 ± 3.2	38.2 ± 3.2	37.1 ± 3.2	0.150	-	-
LA PACS, %	12.8 ± 2.1	14.9 ± 2.7	12.6 ± 2.2	<0.001	0.002	0.935

LAVmax, left atrial maximal volume; LAVmin, left atrial minimal volume; LAVpreA, left atrial pre-A-wave volume; LAEV, left atrial emptying volume; LAEF, left atrial emptying fraction; LA PALS, peak left atrial longitudinal strain; LA PACS, peak left atrial contraction strain. Data are presented as mean ± standard deviation.

Table 4 summarizes exercise-induced changes in RA echocardiographic parameters. There were no significant differences in any RA measures between stage 1 and stage 3. In contrast to LA morphological remodeling, female athletes showed a significantly higher RAVmax index (31.3 ± 6.8 vs. 35.0 ± 7.0 ml/m^2^; *p* = 0.008), along with the RAVpreA index (20.5 ± 5.7 vs. 25.3 ± 6.1 ml/m^2^; *p* < 0.001), RA total EV index (16.9 ± 4.7 vs. 18.7 ± 4.9 ml/m^2^; *p* = 0.030), and RA active EV index (5.9 ± 2.8 vs. 9.0 ± 4.4 ml/m^2^; *p* < 0.001) after the marathon run. Sixty-nine percent of our athletes exceeded the upper limit of 3D RAVmax at rest, whereas the number of women with RA enlargement after the race equaled 94% (*p* = 0.070, stage 1 vs. stage 2). At stage 3, RA dimensions exceeded the upper limit in 75% of runners (*p* = 0.694, stage 1 vs. stage 3). RA contractile function, namely, RA active EF (27.7 ± 8.6 vs. 35.0 ± 12.1%; *p* = 0.014) increased post race, while RA reservoir and passive EF did not differ remarkably between stages. The aforementioned 3D RA differences were accompanied by a significant increment in RA PACS (13.8 ± 1.8 vs. 15.6 ± 2.5%; *p* = 0.016), as assessed by 2D STE.

**TABLE 4:** Right atrial parameters assessed by three-dimensional and two-dimensional speckle tracking echocardiography in female amateur athletes.

Parameter	Female (N = 16)			ANOVA *p*-Value	Post-Hoc *p*-Value	
	Stage 1	Stage 2	Stage 3		S1 vs. S2	S1 vs. S3
RAVmax index, mL/m^2^	31.3 ± 6.8	35.0 ± 7.0	31.1 ± 5.9	0.002	0.008	0.986
RAVmin index, mL/m^2^	14.4 ± 4.0	16.4 ± 3.8	14.3 ± 3.4	0.039	0.081	0.983
RAVpreA index, mL/m^2^	20.5 ± 5.7	25.3 ± 6.1	20.5 ± 4.8	<0.001	<0.001	0.999
RA total EV index, mL/m^2^	16.9 ± 4.7	18.7 ± 4.9	16.8 ± 4.7	0.015	0.030	1.000
RA passive EV index, mL/m^2^	10.5 ± 3.2	9.9 ± 2.8	10.5 ± 3.0	0.795	-	-
RA active EV index, mL/m^2^	5.9 ± 2.8	9.0 ± 4.4	6.0 ± 2.5	<0.001	<0.001	0.996
RA total EF, %	53.9 ± 8.3	53.7 ± 7.7	53.5 ± 8.4	0.984	-	-
RA passive EF, %	33.7 ± 9.1	29.5 ± 7.0	33.0 ± 6.7	0.066	-	-
RA active EF, %	27.7 ± 8.6	35.0 ± 12.1	27.9 ± 7.8	0.007	0.014	0.994
RA PALS, %	37.6 ± 3.6	36.4 ± 3.6	37.6 ± 3.2	0.224	-	-
RA PACS, %	13.8 ± 1.8	15.6 ± 2.5	13.9 ± 1.6	0.010	0.016	0.972

RAVmax, right atrial maximal volume; RAVmin, right atrial minimal volume; RAVpreA, right atrial pre-A-wave volume; RAEV, right atrial emptying volume; RAEF, right atrial emptying fraction; RA PALS, peak right atrial longitudinal strain; RA PACS, peak right atrial contraction strain. Data are presented as mean ± standard deviation.

Taking RA size into consideration, we divided the study participants into two groups according to the presence of RA dilatation at baseline. In athletes with RA volumes above upper limits at rest (N = 11), a significant post-exercise RV dilatation (RVEDV; 51.9 ± 9.5 vs. 63.2 ± 8.2 ml/m^2^; *p* = 0.012) and reduction in function (RVEF; 59.3 ± 7.4 vs. 54.8 ± 5.7%; *p* = 0.027), with concomitant decrease in the LV dimensions (LVEDV; 69.9 ± 6.5 vs. 59.1 ± 9.2 ml/m^2^; *p* = 0.025), was observed. Both RA and LA volumes did not differ remarkably between stages, while RA active EF increased after the marathon (38.7 ± 8.9 vs. 46.6 ± 10.4%; *p* = 0.007). In the group without atrial enlargement before the race, there were no significant post-exercise changes in any of the studied parameters.

**FIGURE 4:**
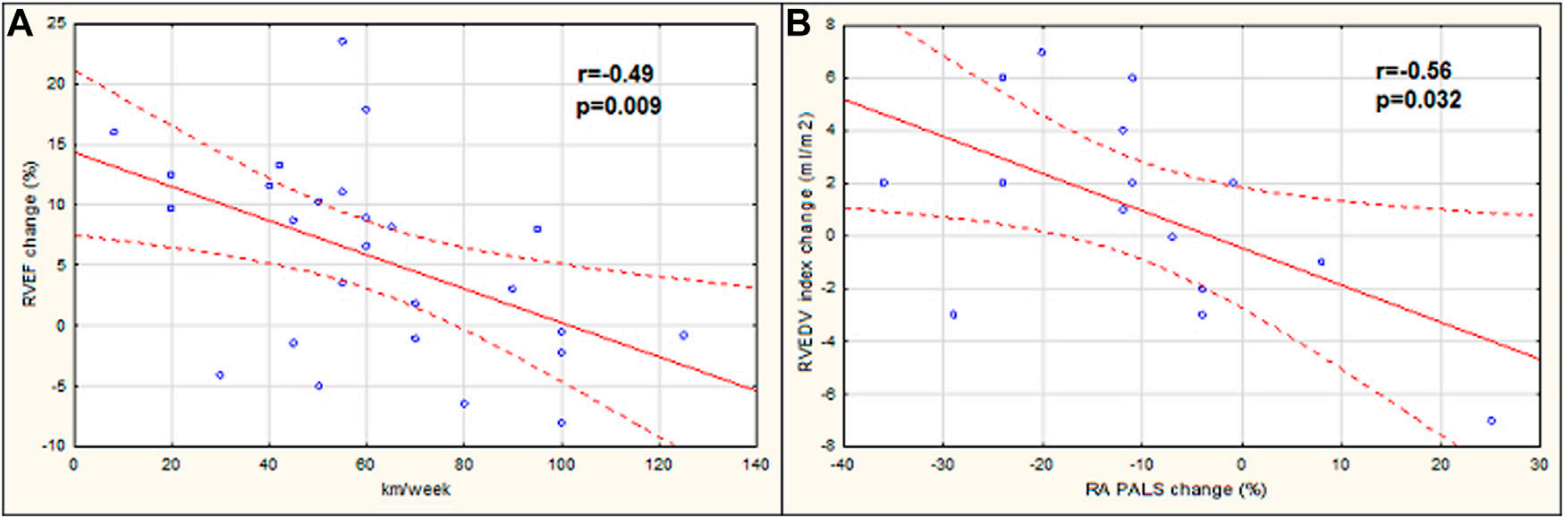
Correlation between the distance of running per week with the right ventricular ejection fraction (RVEF) change **(A)**, the peak right atrial longitudinal strain (RA PALS) change with the right ventricular end-diastolic volume (RVEDV) index change **(B)**. RVEF change, the difference in RVEF stage 1 vs. stage 2; RA PALS change, the difference in RA PALS stage 1 vs. stage 2; RVEDV index change, the difference in RVEDV index stage 1 vs. stage 2.

When analyzing relations between the studied parameters, hours of training per week were positively related to the age of marathoners (r = 0.52, *p* = 0.006), while the distance of running per week inversely correlated with the exercise-induced change of RVEF (r = −0.49, *p* = 0.009) (Figure 4A). The post-race increase in RVEDV was negatively associated with RA PALS change (r = −0.56, *p* = 0.032) (Figure 4B).

## Discussion

The present study provides a comprehensive evaluation of exercise-induced biatrial remodeling in female amateur marathon runners, assessed by 3D echocardiography and 2D STE. We proved that endurance exercise alters echocardiographic parameters of both atrial morphology and function in amateurs, similar to previously described alternations in elite athletes. Although numerous studies have already investigated the effect of marathon running on cardiac structure and function (Kaleta et al., 2017), most are restricted to male athletes or concern resting conditions, with limited data on acute remodeling in female counterparts (Pelliccia et al., 1996; D'Ascenzi et al., 2014).

Women, however, exhibit different cardiac adaptations to exercise than men (Di Paolo and Pelliccia, 2007). There are several biochemical, physiological, and psychological factors that determine sex-dependent cardiac response (Colombo and Finocchiaro, 2018). Women, on average smaller, have lower mean body mass, different autonomic tone, and hormonal profile than men. While taking the hormonal system into consideration, testosterone in men directly stimulates myocardial hypertrophy, while estrogens in women inhibit this process with an opposed effect on cardiac remodeling. According to recent reports, female endurance athletes adapt primarily by increasing ventricular dimensions rather than wall thickness (Pelliccia et al., 1996). It has been proved that the right chambers of the heart are particularly altered. Indeed, female athletes show increased RV cavity size, along with RA dimensions, in comparison to non-athlete participants in baseline conditions (Pelliccia and Adami, 2017). In individuals without cardiovascular disease, RVEF is higher in women than men, which may allow sportswomen to better tolerate exercise-induced RV dilatation. Finally, female athletes are less prone to sudden cardiac death (SCD) mainly because of lower sympathetic activation during endurance training and hormonal protection (Rajan et al., 2021).

To the best of our knowledge, our study is the first one among Polish female amateur athletes that evaluates the acute effect of marathon running on biatrial performance by using 3D echocardiography and 2D STE. In our relatively small study group, we noticed a significant increase in RA size, with no relevant decrease in LA dimensions after the race. Indeed, acute dilatation of RA and RV, but not of LA and LV, has been demonstrated in amateur runners immediately after the marathon race (Trivax et al., 2010; Lewicka-Potocka et al., 2022). In our recent research, which included female marathon runners (Lasocka et al., 2021), we observed a significantly greater number of female athletes meeting the ECG criteria for RA enlargement after the run compared to baseline, with no difference in the incidence of LA enlargement between the stages. Wilhelm et al. (2012a, 2012b) examined the change in proatrial natriuretic peptides before and after the marathon. A remarkable increase of the cardiac biomarker postrace was observed, both in professional and amateur runners, which might reflect higher propensity of exercise-induced atrial dilatation in the study groups.

Along with morphological changes, the atrial functional parameters also evolve with increasing effort in athletes. The application of atrial phasic volumes and strain analysis enables better characterization of myocardial deformation of the atria, distinguishing pathological dysfunction from physiological atrial adaptation to exercise. Most of the studies compare atrial performance and sedentary controls in athletes under resting conditions. According to Lakatos et al. (2020), elite sportsmen had lower reservoir and contractile function, as assessed by 3D LA total EF and LA active EF, while LA passive EF did not differ between the groups. Similar outcomes were described in another study on 3D volumetric changes (Nemes et al., 2017). When taking 2D STE into consideration, a recent analysis revealed that reservoir and contraction strains of either RA or LA were decreased in athletes at rest than in non-sportive subjects (Gabrielli et al., 2014). These findings are consistent with those of Sanchis et al. (2017), who found lower biatrial deformation values in athletes, with significantly higher RA and LA contractile function in women than men at rest.

However, data are scarce regarding atrial functional measures during or directly after endurance training. In our study, female amateur runners showed a remarkable increase in LA active EF and PACS post-race, while LA reservoir and conduit function remained unchanged. In RA, prolonged exercise induced a significant increment in atrial contractile function, as assessed by 3D and 2D STE parameters. When it comes to volumetric data, Wright et al. (2015) showed that both LA passive and reservoir EV did not change during moderate exercise, while LA pump function significantly increased. Similar outcomes were obtained by Cavigli et al. (2022), who described increased LA PACS directly after an ultra-marathon run, without changes in reservoir or conduit function using strain analysis. These observations, based also on female athletes, agree with our findings and could be explained by LA contribution to maintain LV filling according to the Frank–Starling mechanism. Although other authors confirmed the post-exercise increment in LA PACS (Oxborough et al., 2010; Gabrielli et al., 2014), all of these studies are based on the male population. The extent of atrial adaptation to exercise changes during the training period Sanz-de la Garza et al. (2016) showed an increase in atrial reservoir and contractile function in distances up to 35 km, with a further decrease post 56 km. Only a small group of post 56 km runners proved to increase atrial contractile function. That demonstrates different atrial responses to the training stimulus among athletes.

Atrial remodeling might be a physiologic adaptation to volume overload, permitting a greater volume delivery and increased cardiac output. The rise of preload and afterload during exercise seems to particularly involve the RV in terms of chamber dilatation and lower deformation, without impairment in LV function (La Gerche et al., 2012), which can be a result of increased pulmonary pressure during the race. Indeed, we noticed a significant increase in RV dimensions and concomitant reduction in RV function among study participants after the marathon. In the recent analysis of male amateur athletes, Lewicka-Potocka et al. (2022) also observed a transiently enlarged RV with reduced contractility, as assessed by a decrease in RV radial shortening. The reduction in RV systolic function correlated with the intensity of the preparation period, in the form of a “U-shaped curve.” Both athletes with very minimal training and those with more than 47 km running per week presented a greater decrease in RV contractility post race. On the contrary, in our study, the greater training volume per week was associated with lower RV dysfunction, especially among participants with more than 60 km running per week. These findings are supported by Neilan et al. (2006). In the research on 60 non-elite marathon runners, both men and women, the training mileage of the study participants was inversely related to RV contractility, pulmonary pressures, and level of cardiac biomarkers. Compared with athletes training above 45 miles per week, those who run less than 35 miles per week demonstrated increased pulmonary pressures, greater RV dysfunction, and increased cardiac troponin T level and N-terminal pro-brain natriuretic peptide. As reported, the extent of the preparation period determines cardiac response to endurance training, such as a marathon run, and should be individually counted.

Secondary to RV changes, the impairment of RA may also be observed. When ventricular afterload increases, atrial reservoir function is primarily affected. In contrast, atrial contractile function is maintained or even increased. Although in our research, RA reservoir function did not differ significantly between stages, in athletes with a greater post-race increase in RVEDV, a lower increment in RA PALS was observed. As all of the abnormalities were transient and not reported in the control examination, they should be classified as physiological aspects of the “athlete’s heart” rather than pathological alternations.

However, repetitive stretching of the atria may predispose to chronic structural changes in response to the recurrent volume overload and excessive cardiac strain (Pluim et al., 2000; Maron and Pelliccia, 2006). Król et al. (2016) examined 114 international-level rowers and described LA enlargement in nearly half of the enrolled athletes (43%), with higher frequency in men than in women (52.5% vs. 32.1%; *p* < 0.05), while Wilhelm et al. (2012) demonstrated an independent effect of repeated marathon running on biatrial size, with larger RA and LA volumes in endurance athletes compared to non-marathon runners. In subjects who participated in six or more marathons, right and left atrial enlargements were present in 60% and 74% of athletes, respectively. In our study, at baseline, 48% of the study participants exceeded the upper limit of LA volume, while RA enlargement was present in 69% of women. Moreover, after dividing the study participants according to the initial presence of RA dilatation, we noticed a significant post-exercise RV dilatation and reduction in systolic function, along with decrease in the LV dimensions in those with RA size above the upper limits at rest. Therefore, the baseline increase in atrial volumes may indicate a higher prevalence of exercise-induced changes, which, over a long time period, may result in persistent cardiac remodeling.

Atria working at higher wall stress are more susceptible to the development of atrial fibrosis, along with incidental atrial arrhythmia (Gabrielli et al., 2014). Recent reports have shown a relationship between endurance training and AF (Abdulla and Nielsen, 2009; Aizer et al., 2009; Mont et al., 2009). According to the latest analysis, reduced strain values of LA, especially reservoir strain, are strong identifiers of AF (Sørensen et al., 2021). It has been proven that regardless of athletic status, both reservoir and contractile strains are lower in those with AF. The possible mechanisms explaining the association remain speculative. Atrial remodeling, vagal tone, and atrial ectopic triggers are found to contribute to increased incidence of AF in non-elite endurance athletes (Wilhelm et al., 2011). Although our post-race cardiac monitoring did not reveal any increase in any form of supraventricular arrhythmia, this observation cannot correlate to possible arrhythmias that occurred during the race itself. RA dilatation, which was high both at rest and after the run in our study group, could be the substrate for arrhythmias (Mont et al., 2009), especially in the long-term follow-up. Further research is needed to fully understand the mechanism underlying AF development in sportsmen.

This study is the first one that assessed and compared atrial remodeling at rest and after a marathon run in female amateur athletes by using 3D echocardiography. Compared with 2D assessment, 3D echocardiography provides more accurate measurements of atria and has superior prognostic ability (Artang et al., 2009; Caselli et al., 2010). Moreover, 3D echocardiography offers an additional capability to predict cardiovascular events (Badano et al., 2016). The availability of 3D reference values will help clinicians identify exercise-induced atrial remodeling and differentiate it from potential atrial dysfunction.

Several limitations of our study should be noticed. First, the study was carried out in a relatively small sample and involved only white female amateur athletes living in the Pomeranian Voivodeship, Poland, limiting its statistical power and generalizability. Moreover, the number of female participants with adequate RA images both at rest and after the run was low because during the marathon in 2018, major attention was given to ventricular function. Second, it lacks a control group of non-amateur marathon runners matched by sex, age, height, and weight, or male amateur runners to detect gender differences in atrial remodeling in resting conditions. In addition, the study focuses on the comparison of cardiac changes before and directly after endurance training, without any data obtained during the exercise itself. Therefore, the observed atrial remodeling may not fully extrapolate to the acute exertion phase. Finally, there is a lack of long-time follow-up, which makes it impossible to confirm whether the observed atrial performance predisposes to a higher prevalence of AF among study participants in the future.

## Conclusion

Long-term physical training promotes biatrial remodeling in female non-elite athletes, with more pronounced changes in the RA. After the marathon, a significant dilatation of both RV and RA and a concomitant decrease in RV contractility were observed. These changes were especially present in women with RA enlargement in baseline conditions. When analyzing left heart chambers, LV dimensions decreased post race, and no relevant reduction in LA size was observed, without any functional impairment. Post race, the active contraction phase of both atria increased. However, considering atrial reservoir function, greater RV dilatation was observed post race and lower increase in RA PALS was detected. Although the observed changes were proved reversible, the long-term consequences of repeated strenuous training remain to be clarified. A better understanding of atrial adaptation to a marathon run among amateurs is clinically relevant for clarifying the risk of exercise-induced arrhythmias. Moreover, our study suggests that appropriate preparation before a marathon is important to protect against greater RV dysfunction.

## Data Availability

The original contributions presented in the study are included in the article/Supplementary Material; further inquiries can be directed to the corresponding author.
