# Supplementary material for: Exercise-Induced Atrial Remodeling in Female Amateur Marathon Runners Assessed by Three-Dimensional and Speckle Tracking Echocardiography
Source: Front Physiol. 2022 Jul 4;13:863217. doi: 10.3389/fphys.2022.863217 (PMC9289460; doi:10.3389/fphys.2022.863217)
Supplement: Supplementary file 1 [file Table1.DOCX]

| **Parameter** | **Generalized eta squared** | **The Kendall’s W** | **Power** |
| --- | --- | --- | --- |
| LVEDV index, mL/m2 | 0.165 | - | 0.165 |
| LVESV index, mL/m2 | - | 0.430 | 0.835 |
| RVEDV index, mL/m2 | 0.117 | - | 0.113 |
| RVESV index, mL/m2 | - | 0.430 | 0.836 |
| RVEF, % | 0.100 | - | 0.095 |
| RVFAC, % | 0.161 | - | 0.176 |
| LA active EF, % | - | 0.100 | 0.108 |
| LA PACS, % | 0.157 | - | 0.218 |
| RAVmax index, mL/m2 | 0.077 | - | 0.071 |
| RAVpreA index, mL/m2 | 0.142 | - | 0.124 |
| RA total EV index, mL/m2 | - | 0.241 | 0.283 |
| RA active EV index, mL/m2 | 0.170 | - | 0.160 |
| RA active EF, % | - | 0.281 | 0.373 |
| RA PACS, % | 0.142 | - | 0.119 |

**Supplementary table 1.** The power calculation of the studied echocardiographic parameters with statistically significant post-marathon differences.

LVEDV, left ventricular end-diastolic volume; LVESV, left ventricular end-systolic volume; RVEDV, right ventricular end-diastolic volume; RVESV, right ventricular end-systolic volume; RVEF, right ventricular ejection fraction; RVFAC, right ventricular fractional area change; LAEF, left atrial emptying fraction; LA PACS, peak left atrial contraction strain; RAVmax, right atrial maximal volume; RAVpreA, right atrial pre-A-wave volume; RAEV, right atrial emptying volume; RAEF, right atrial emptying fraction; RA PACS, peak right atrial contraction strain.
